# Daptomycin Resistance in Enterococci Is Associated with Distinct Alterations of Cell Membrane Phospholipid Content

**DOI:** 10.1371/journal.pone.0043958

**Published:** 2012-08-27

**Authors:** Nagendra N. Mishra, Arnold S. Bayer, Truc T. Tran, Yousif Shamoo, Eugenia Mileykovskaya, William Dowhan, Ziqiang Guan, Cesar A. Arias

**Affiliations:** 1 Division of Infectious Diseases, Los Angeles Biomedical Research Institute at Harbor-University of California Los Angeles Medical Center, Torrance, California, United States of America; 2 David Geffen School of Medicine at University of California Los Angeles, Los Angeles, California, United States of America; 3 Division of Infectious Disease, Laboratory for Antimicrobial Research, University of Texas Medical School at Houston, Houston, Texas, United States of America; 4 University of Houston College of Pharmacy, Houston, Texas, United States of America; 5 Department of Biochemistry and Cell Biology, and Department of Ecology and Evolutionary Biology, Rice University, Houston, Texas, United States of America; 6 Department of Biochemistry and Molecular Biology, University of Texas Medical School at Houston, Houston, Texas, United States of America; 7 Department of Biochemistry, Duke University Medical Center, Durham, North Carolina, United States of America; 8 Molecular Genetics and Antimicrobial Resistance Unit, Universidad El Bosque, Bogota, Colombia; Institut Pasteur, France

## Abstract

**Background:**

The lipopeptide antibiotic, daptomycin (DAP) interacts with the bacterial cell membrane (CM). Development of DAP resistance during therapy in a clinical strain of *Enterococcus faecalis* was associated with mutations in genes encoding enzymes involved in cell envelope homeostasis and phospholipid metabolism. Here we characterized changes in CM phospholipid profiles associated with development of DAP resistance in clinical enterococcal strains.

**Methodology:**

Using two clinical strain-pairs of DAP-susceptible and DAP-resistant *E. faecalis* (S613 vs. R712) and *E. faecium* (S447 vs. R446) recovered before and after DAP therapy, we compared four distinct CM profiles: phospholipid content, fatty acid composition, membrane fluidity and capacity to be permeabilized and/or depolarized by DAP. Additionally, we characterized the cell envelope of the *E. faecium* strain-pair by transmission electron microscopy and determined the relative cell surface charge of both strain-pairs.

**Principal Findings:**

Both *E. faecalis* and *E. faecium* mainly contained four major CM PLs: phosphatidylglycerol (PG), cardiolipin, lysyl-phosphatidylglycerol (L-PG) and glycerolphospho-diglycodiacylglycerol (GP-DGDAG). In addition, *E. faecalis* CMs (but not *E. faecium*) also contained: ***i***) phosphatidic acid; and ***ii***) two other unknown species of amino-containing PLs. Development of DAP resistance in both enterococcal species was associated with a significant decrease in CM fluidity and PG content, with a concomitant increase in GP-DGDAG. The strain-pairs did not differ in their outer CM translocation (flipping) of amino-containing PLs. Fatty acid content did not change in the *E. faecalis* strain-pair, whereas a significant decrease in unsaturated fatty acids was observed in the DAP-resistant *E. faecium* isolate R446 (vs S447). Resistance to DAP in *E. faecium* was associated with distinct structural alterations of the cell envelope and cell wall thickening, as well as a decreased ability of DAP to depolarize and permeabilize the CM.

**Conclusion:**

Distinct alterations in PL content and fatty acid composition are associated with development of enterococcal DAP resistance.

## Introduction

Enterococci are leading causes of nosocomial infections in the US [1], causing a variety of life-threatening syndromes such as bacteremic infections (including endocarditis), urosepsis and meningitis, among others. Enterococcal disease occurs frequently in patients that are seriously ill and/or with important degrees of immunosuppression. Two species are responsible for the vast majority of enterococcal infections, *E. faecalis* and *E*. *faecium*. The treatment of such infections is often impacted by the increased prevalence of multidrug resistance in these isolates. Indeed, ampicillin and vancomycin resistance is now present in more than 80% of *E. faecium* isolates [1], making these compounds almost obsolete for the treatment of this pathogen. Moreover, *E. faecium* is one of the “no ESKAPE” pathogens (***E*** *. faecium*, ***S*** *taphylococus aureus*, ***K*** *lebsiella pneumoniae*, ***A*** *cinetobacter baumanii*, ***P*** *seudomonas aeruginosa*, ***E*** *nterobacter* spp.), highlighted by the Infectious Diseases Society of America as nosocomial organisms requiring new therapeutic approaches, both because of their commonality and challenging clinical presentations, but importantly also due to multiple antibiotic resistances [2]. Although most *E. faecalis* isolates remain susceptible to ampicillin, the increased prevalence of resistance to aminoglycosides and vancomycin within this species also limits the therapeutic alternatives, as well as the ability to achieve bactericidal killing of this organism [1], [3].

Daptomycin (DAP) is a lipopeptide antibiotic approved by the Food and Drug Administration (FDA) in 2003 for the treatment of skin and soft tissue infections caused by susceptible Gram-positive organisms and, subsequently in 2006, for *S. aureus* bacteremia and right-sided endocarditis [4]. DAP has potent *in vitro* bactericidal activity against vancomycin-resistant enterococci (VRE), although it does not have an FDA approval for VRE infections. Nonetheless, many clinicians often use this compound for the treatment of severe enterococcal infections, particularly those caused by *E. faecium*. Two retrospective studies have shown that outcomes of patients with serious enterococcal infections treated with DAP were similar to those treated with linezolid (an FDA-approved antibiotic for VRE infections) [5], [6]. However, there are several recent reports of patients failing DAP mono-therapy in association with emergence of DAP resistance during therapy [7], [8], [9].

The mechanism of action of DAP involves a calcium-dependent interaction with the bacterial cell membrane (CM) [10], [11]. The insertion of the drug into the CM causes disruption in its homeostasis that is associated with a leakage of potassium ions from the cytoplasm of the bacterial cell [10]. These alterations of the CM lead to bacterial cell death by mechanisms that are not fully elucidated but are likely to involve alterations in cell division homeostasis [11]. Emergence of DAP non-susceptibility has been described in both *S. aureus* and enterococci. Several genes have been associated with this phenomenon and include: ***i***) genes encoding two or three-component regulatory systems involved in cell envelope homeostasis: *yvqF*-*vraSR* in *S. aureus*, and *liaFSR* in enterococci and *Bacillus subtilis* [12], [13]; ***ii***) genes coding for enzymes involved in CM phospholipid metabolism: *mprF* (multiple peptide resistance factor) [12], [14], [15], *cls* (cardiolipin synthase) [14], *pgsA* (phosphatidylglycerol synthase) [14] in *S. aureus*; *gdpD* (glycerophosphodiesterphosphodiesterase) [13], *cls* [13], [16] in enterococci, and *pgsA* [17] in *B. subtillis*; and ***iii***) genes encoding for β subunits of RNA polymerase in *S. aureus (rpoB/rpoC)* [14], [18].

In the present study, we delineate the following CM characteristics of both two enterococcal clinical strain-pairs (DAP-susceptible and DAP-resistant), each isolated from a patient during failed DAP therapy: ***i***) phospholipid repertoire, ***ii***) fatty acid composition, ***iii***) fluidity, and ***iv***) DAP-induced permeabilization-depolarization profiles.

## Results

### CM phospholipids (PLs) of *E. faecalis* vs. those of *E. faecium*

**Figure 1:**
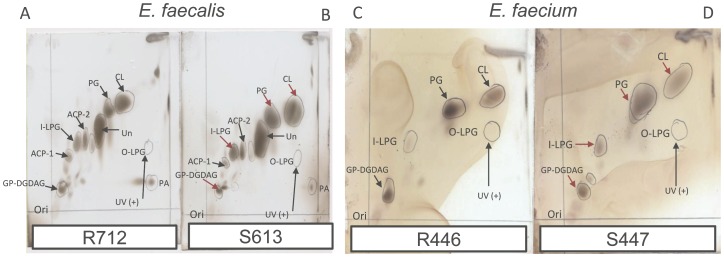
Phospholipid (PL) profiles by 2D-TLC. Phospholipid spots shown in 2D-TLC plates were developed with CuSO4 (100 mg/ml) spray containing 8% phosphoric acid and heated at 180°C. ACP-1 and ACP-2, amino-containing PL-1 and -2; I-LPG, inner CM layer-lysyl-phosphatidylglycerol; O-LPG, outer CM layer lysyl-phosphatidylglycerol; PG, phosphatidylglycerol; CL,cardiolipin; GP-DGDAG, glycerolphospho-diglycodiacylglycerol; PA, phosphatidic acid; Un, unidentified phospholipid. Red arrows indicate the major spots confirmed by LC/ESI-MS/MS analysis. The spot of fluorescamine-labelled O-LPG was detected by UV, indicating flipping of the latter PL to the outer CM.

Each strain-pair had an identical PFGE profile (data not shown). The DAP minimal inhibitory concentrations (MICs) for the *E. faecalis* pair were 1 µg/ml and 12 µg/ml for S613 and R712, respectively. For *E. faecium* S447 and R446, the DAP MICs were 2 µg/ml and 16 µg/ml, respectively. **Table 1** shows the CM PL content of the clinical strain-pairs of *E. faecalis* and *E. faecium*; **Figure 1** shows these PL repertoires as identified on two-dimensional thin layer chromatography (2D-TLC) plates with each major spot subsequently confirmed by liquid chromatography/ electrospray ionization-mass spectrometry/mass spectrometry (LC/ESI-MS/MS) analysis. The CM PLs of the *E. faecium* pair were less complex than those of *E. faecalis*. For example, lysyl-phosphatidylglycerol (L-PG) was the only amino-containing (positively-charged) PL detected in *E. faecium* (**Table 1**), whereas *E. faecalis* CMs contained L-PG plus two additional amino-containing PLs of unknown identity (**Table 1** **,** **Figure 1**). Moreover, the PLs of *E. faecium* were composed mainly of phosphatidylglycerol (PG), cardiolipin (CL) and a glycerophosphoglycolipid identified by LC/ESI-MS/MS as glycerolphospho-diglycodiacylglycerol (GP-DGDAG), whereas phosphatidic acid (PA) and an unidentified non-amino-containing PL were also detected in *E. faecalis* CMs (**Table 1** **,** **Figure 1**).

**Table 1:** Cell membrane phospholipid (PL) composition and asymmetry in the *E. faecalis* and *E. faecium* clinical strain-pairs.

PLs Species	*E. faecalis*		*E. faecium*	
	% of total PL composition and asymmetry		% of total PL composition and asymmetry	
	S613 (DAP-S)	R712 (DAP-R)	S447 (DAP-S)	R446 (DAP-R)
ACP-1	3.39±2.37	5.29±1.05	_	_
ACP-2	4.14±0.99	5.00±1.24	_	_
Inner LPG	6.76±1.06	6.49±1.39	8.12±0.89	6.80±5.25
Outer LPG	7.77±1.08	8.34±1.65	6.27±4.77	8.91±3.87
Total LPG	14.53±1.16	14.83±2.96	14.39±5.66	15.71±4.56
Total ACP	22.06±2.93	25.12±3.81	_	_
PG	27.90±4.43	20.11±3.38 *	33.77±2.65	14.65±2.38**
CL	31.28±1.63	29.17±3.70	38.99±1.30	46.55±5.51
GP-DGDAG	5.20±1.32	8.07±1.36 *	12.84±1.71	23.08±8.36**
PA	6.03±1.40	7.06±2.39	_	_
Un	7.53±1.27	10.47±1.95 *	_	_

PLs, phospholipid; ACP-1, amino-containing phospholipid 1; ACP-2, amino-containing phospholipid 2; LPG, lysyl-phosphatidylglycerol; PG, phosphatidylglycerol; CL, cardiolipin; GP-DGDAG, glycerolphospho-diglycodiacylglycerol; PA, phosphatidic acid; Un, unidentified phospholipid.

* P<0.05 in relation to S613; **P<0.01 in relation to S447. Statistically significant values are underlined.

Interestingly, in both enterococcal pairs, a significant decrease in PG content was observed in the DAP-resistant variants (**Table 1**), with the reduction in PG being quite pronounced in *E. faecium* R446 (∼50% vs the parental S447 strain). This decrease in PG content in both DAP-resistant strains (*E. faecalis* R712 and *E. faecium* R446) was accompanied by a significant increase in the glycerophosphoglycolipid GP-DGDAG content as compared to their parental strains. Also, in *E. faecalis* R712, there was a concomitant increase in an unidentified negatively-charged PL species (**Table 1**). We were unable to detect a significant difference in the CL content of the DAP-resistant derivatives of either *E. faecalis* or *E. faecium* (R712 and R446, respectively) when compared with their DAP-susceptible parental isolates (**Table 1**) although, a trend towards increased CL content was observed in *E. faecium* R446 vs S447 (p = 0.067). Another important finding was that, in contrast to DAP resistance in *S. aureus* [19], the amount of amino-containing PLs in the inner vs. outer CM leaflet (asymmetry) did not change significantly between the DAP-susceptible and DAP-resistant clinical strain-pairs of either *E. faecalis* or *E. faecium* (**Tables 1**).

### CM fluidity in enterococcal strain pairs

**Table 2** shows that the DAP-resistant derivatives of both *E. faecalis* and *E. faecium* (R712 and R446, respectively) had a significantly higher polarization index value as compared with their respective parental DAP-susceptible isolates (S613 and S447, respectively), indicating less fluid (more rigid) membranes.

**Table 2:** Polarization index values as indicators of CM fluidity in the enterococcal clinical strain-pairs.

*E. faecalis*	S613 (DAP-S)	R712 (DAP-R)	*E. faecium*	S447 (DAP-S)	R446 (DAP-R)
	0.281±0.018	0.329±0.011*		0.287±0.003	0.303±0.002*

* p<0.005 in relation to the susceptible isolate.

### Fatty acid compositional analysis

**Table 3** shows the CM fatty acid composition of the enterococcal clinical strain-pairs. We found no statistically significant difference in the patterns of saturated fatty acids (SFAs), unsaturated fatty acids (UFAs), and cyclic fatty acid (CFA) profiles between the *E. faecalis* S613 (DAP-susceptible) and its DAP-resistant derivative (R712). In contrast, a decrease in the proportion of total UFAs and an increase in CFAs were observed in the DAP-resistant *E. faecium* R446 as compared to its DAP-susceptible parental strain, S447 (P = 0.0121 and 0.044, respectively). This decrease in total UFAs appeared to be driven by a substantial reduction in the major UFA species, C18:1ω7c (**Table 3**).

**Table 3:** Fatty acid composition of the *E. faecalis* and *E. faecium* clinical strain-pairs.

Nature of Fatty Acid	*E. faecalis*		*E. faecium*	
	% of fatty acid composition		% of fatty acid composition	
	S613 (DAP-S)	R712 (DAP-R)	S447 (DAP-S)	R446 (DAP-R)
**Saturated fatty acids (SFA)**				
**• 14:0**	6.68±0.05	5.45±0.24	7.76±0.16	8.62±0.03
**• 16:0**	24.78±0.72	22.58±0.52	26±0.03	26±0.11
**• 18:0**	5.95±0.36	5.55±0.26	4.67±0.12	5.3±0.20
**• Total SFA**	**37.4±1.03**	**33.58±1.03**	**38.37±0.07**	**39.705±0.28**
**Unsaturated fatty acids (UFA)**				
**• 16:1ω7c**	14.83±0.54	15±0.11	12.5±0.19	14.2±0.23 *
**• 18:2ω6,9c**	5.645±0.27	4.93±0.15	6.42±0.18	5.99±0.33
**• 18:1ω9c**	2.135±0.19	2.43±0.09	4.15±0.23	3.12±0.12
**• 18:1ω7c**	28.315±0.13	33.84±1.1	34.2±0.02	28.8±0.26 *
**• Total UFA**	**50.92±0.21**	**56.2±0.96**	**57.285±0.19**	**52.09±0.04** *
**Cyclic fatty acid (CFA)**				
**• 19:0 cyclo ω8c**	9.33±0.61	7.75±0.01	3.26±0.071	6.9±0.403 *

* p<0.05 in relation with S447. Statistically significant values are underlined.

### Ultrastructural cell envelope alterations and cell wall thickness

**Figure 2:**
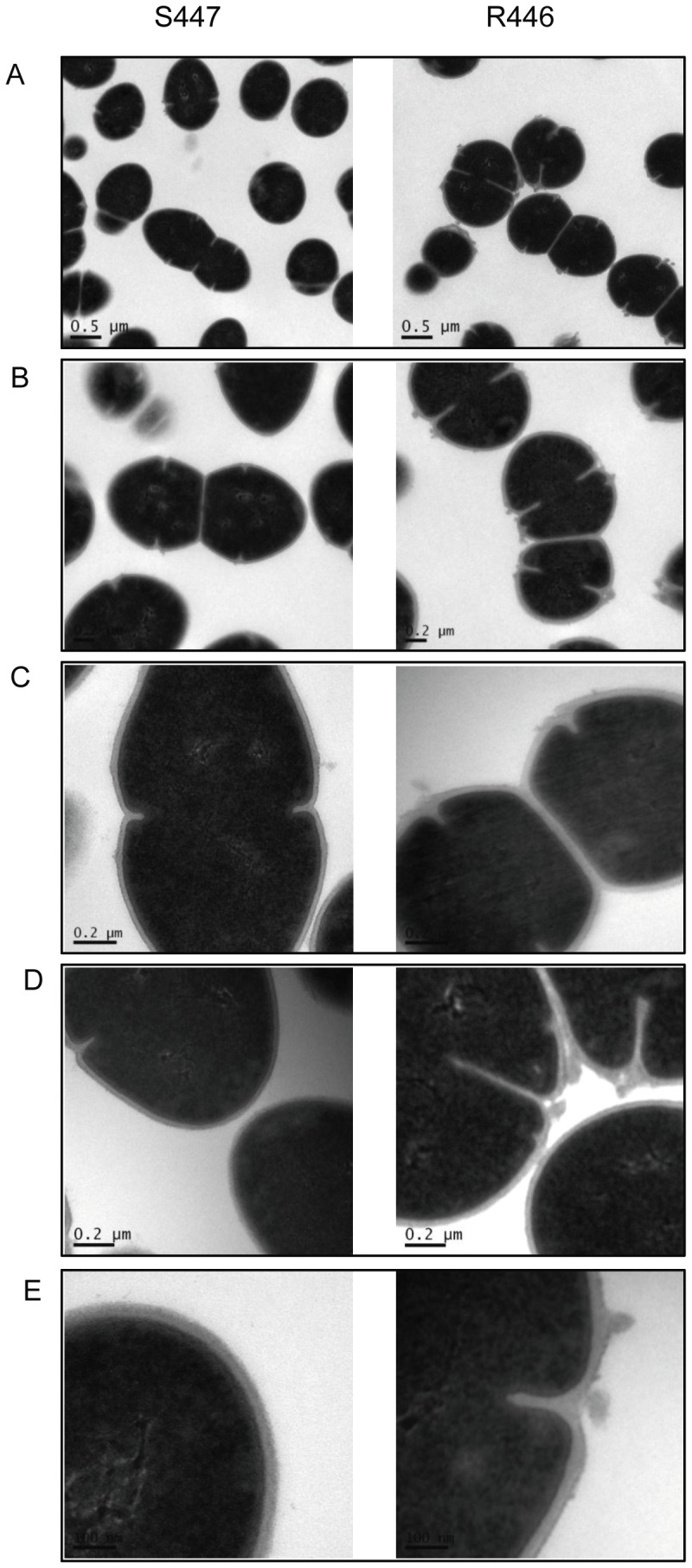
Transmission electron microscopy of cell envelope of *Enterococcus faecium* isolates (daptomycin-susceptible, S447 and daptomycin-resistant, R446). At high magnification, cells in the S447 isolate have multiple septa with easily detectable separation (Panels A and B) and cell envelope of S447 have smooth appearance (panels C, D and E). In contrast, cells from the R446 isolate are in close proximity with each other (panel A). Dividing cells of the R446 isolate tend to have aberrant placement of septa (arrows) and cell envelopes appeared to be altered at forming septa (panels B and D). Localized protrusions of the cell envelope, usually within close proximity to a septal structure, are observed in cells of R446 (panels C, D, and E).

We previously observed several unique ultrastructural cell envelope alterations in association with DAP resistance in *E. faecalis* [13]. Hence, we similarly characterized the cell envelope of our clinical strain-pair of *E. faecium* using transmission electron microscopy. Similar to the above prior *E. faecalis* analyses, we confirmed a significant increase in the thickness of the cell wall in the DAP-resistant *E. faecium* R446 as compared to its parental DAP-susceptible strain, S447 (35.1±7.1 nm vs. 25.6±3.8 nm, respectively, P<0.001). Moreover, we also found impressive disruptions in the architecture of the cell envelope of the DAP-resistant *E. faecium* strain, the most striking of which included the appearance of cell envelope protrusions (**Figure 2**), similar to those we previously described in DAP-resistant *E. faecalis* [13].

### Relative cell surface positive charge and DAP-mediated CM permeabilization-depolarization profiles

Similar to data generated previously in our *E. faecalis* clinical strain-pair [13], emergence of resistance to DAP in *E. faecium* was associated with an increase in the relative net surface positive charge, reflected by a significant decrease in the percentage of surface-bound cytochrome c (∼7% in *E. faecium* R446 vs ∼17% in S447; P = 0.0016).

**Figure 3:**
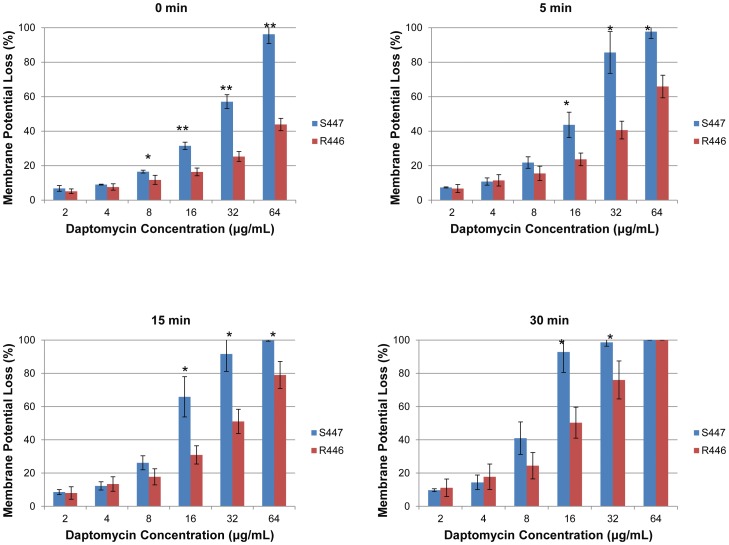
CM depolarization by DiSC_3(5)_ after daptomycin exposure of daptomycin-susceptible (S447) and daptomycin-resistant (R446) *Enterococcus faecium.* Data represent the mean (±SD). * p<0.05; ** p<0.001.

**Figure 3** quantifies the ability of DAP to depolarize the CM of the *E. faecium* clinical-strain pair. There was a statistically significant reduction in the ability of DAP to depolarize the CM in DAP-resistant *E. faecium* R446 as compared to its DAP-susceptible parental strain, S447. This difference was observed immediately after exposure to DAP (time 0), especially at concentrations >8 µg/ml. The reduction in DAP-mediated CM depolarization observed in R446 was maintained at 5, 15 and 30 min after DAP exposure, and was most prominent at DAP concentrations of 16 and 32 µg/ml. Of note, these differences in depolarization profiles between the DAP-susceptible and DAP-resistant strains were less evident at the highest DAP concentrations tested (32 and 64 µg/ml) in a time-dependent manner.

**Figure 4:**
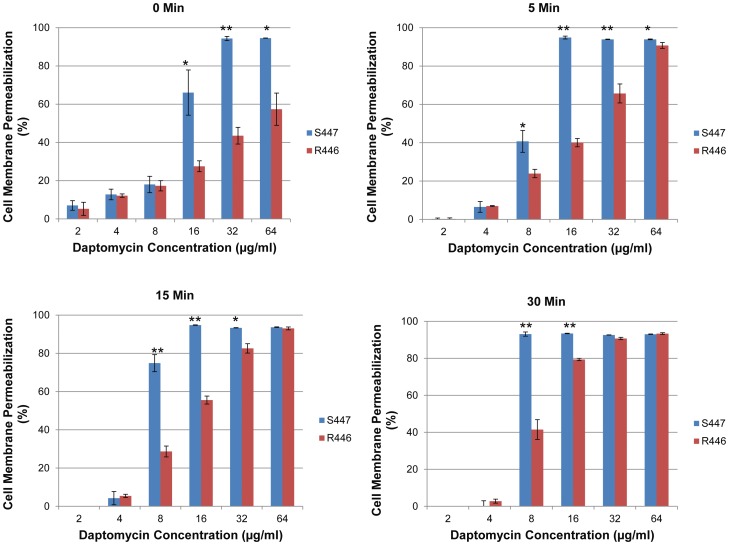
CM permeabilization by LIVE/DEAD BacLight after daptomycin exposure of daptomycin-susceptible (S447) and daptomycin-resistant (R446) *E. faecium*. Data represent mean (±SD); * p<0.05, ** p<0.001.

Similarly, we observed a substantial decrease in the ability of DAP to permeabilize the CM of the DAP-resistant *E. faecium* R446 vs. the DAP-susceptible parental isolate (S447) (**Figure 4**). There was a reduction in DAP-dependent CM permeabilization of *E. faecium* R446 vs S447, particularly at DAP concentrations of 8 and 16 µg/ml after 5 minutes of exposure to daptomycin (**Figure 4**).

## Discussion

DAP is one of the few antibiotics that exhibit *in vitro* bactericidal activity against enterococci, including those that are vancomycin- and ampicillin-resistant [20]. This antibiotic is now commonly used “off-label” for the treatment of serious enterococcal infections due to the lack of other therapeutic alternatives. A challenging issue for the use of DAP against enterococci is the emergence of resistance during therapy. Indeed, several cases of patients infected with *E. faecalis* and *E. faecium* who developed resistance during the course of DAP treatment have now been documented [7], [8], [9]. Furthermore, enterococci are less susceptible to DAP *in vitro* than staphylococci, and this relatively reduced ability of DAP to kill enterococci may contribute to the development of DAP-resistance *in vitro*, and subsequent failure during DAP therapy of serious infections.

The mechanism of action of DAP involves interactions with the bacterial CM in a calcium-dependent manner [11]. We have previously shown that the development of DAP-resistance in a clinical-strain pair of *E. faecalis* was associated with important ultrastructural changes in the cell envelope, including an increase in cell-wall thickness, as well as notable perturbations in cell surface charge and a reduced ability of DAP to depolarize and permeabilize the CM [13]. In the current work, we extended the above observations to a clinical strain-pair of *E. faecium* and, most importantly, focused our studies on a comparative analysis of the CM PL profile of both clinical-strain pairs. A number of interesting themes emerged from our investigations. *Firstly*, as we observed previously in *E. faecalis*, development of DAP resistance in *E. faecium* was associated with marked structural and functional changes in the cell envelope, and in the ability of DAP to alter the CM integrity. Thus, development of DAP resistance in *E. faecium* was accompanied by structural perturbations within the cell envelope (e.g., peri-septal CM protrusions) and in the cell wall (thickening). Also, biophysically, the CMs of both DAP-resistant enterococci became less fluid. Our findings confirm that structural alterations of the cell envelope and changes in the biophysical properties of the CM are strongly associated with the mechanism of DAP resistance, as previously described in *S. aureus* [13], [19] and *B. subtillis* [11].

*Secondly*, detailed analysis of CM PL content in both clinical strain-pairs revealed important differences between *E. faecalis* and *E. faecium*. Indeed, whereas in *E. faecium*, the only amino-containing PL detected was L-PG (similar to *S. aureus*) [19], *E. faecalis* CMs contained at least three amino-PLs; one was identified as L-PG (∼15% of total PLs), with two other amino-PLs of unknown identity. To our knowledge, a study that comprehensively characterized the amino-containing PLs of *E. faecalis* was published over 40 years ago [21], and provided compelling evidence that lysyl-, alanyl- and arginyl-containing PLs were present in their CM extracts. A recent study also noted the presence of alanyl- and arginyl-containing PLs in their membrane lipid extract [22]. Although our experimental growth conditions for PL extraction were different from those used in the former study (BHI vs medium containing 1% tryptone, 0.5% yeast extract, 0.5% dipotassium phosphate and 1% glucose, respectively), it is tempting to speculate that the other amino-containing PLs identified in our *E. faecalis* strain-pair are likely to represent alanyl-PG and arginyl-PG. Of interest, we did not find any significant difference in the amino-containing PL content or L-PG “flipping” between the DAP-resistant *E. faecalis* R712 compared to its parental DAP-susceptible S613. In contrast, in our *E. faecium* strain-pair, a trend towards increased content of L-PG in the outer CM of the DAP-resistant isolate was observed, although this did not quite reach statistical significance. Our findings suggest that differences in content and distribution (“flipping”) of amino-containing PLs are not a major determinant of DAP resistance in enterococci. This is in contrast to data from *S. aureus* where increased L-PG translocation to the outer CM leaflet (likely mediated by gain-in-function mutations in the CM lipid-modifier protein, MprF) appears to play a pivotal role in DAP-R in selected strains [23], [24].

*Thirdly*, a striking finding in our studies was that the *in vivo* development of DAP resistance in enterococci was associated with a significant decrease in PG content. Such striking reductions in CM PG have been previously associated with resistance to DAP and other cationic antimicrobial peptides in Gram-positive bacteria [14], [25]. For example, using an *in vitro* passage strategy for developing DAP-resistance, Hachmann et al. [17] demonstrated that development of DAP resistance was associated with a marked decrease in CM PG content in a laboratory strain of *B. subtilis*, although changes in other CM PLs were not described. The reduction in PG was correlated with the presence of a single nucleotide polymorphism (A64V) in *pgsA*, a gene encoding a key PG synthase. Of interest, mutations in *pgsA* have also been recently associated with DAP-resistance in *S. aureus* [14]. Similarly, characterization of *E. faecium* mutants resistant to the class II bacteriocin, mundticin KS (a 43 amino acid peptide produced by *E. mundtii* and active against *E. faecium*), was associated with significant reductions in PG (and also CL) [25]. PG is an abundant anionic PL of bacterial CMs in both Gram-positive and Gram-negative bacteria [26]. In *E. coli*, the balance of zwitterionic and acidic (negatively-charged) PLs is the result of the formation of PG, which is tightly coupled with the regulation of the synthesis of other PLs [26]. Maintaining the PL balance is crucial for the homeostasis of several bacterial processes including CM protein topology [27] and cell division [28], among others. Interestingly, we previously showed that a codon deletion in a gene encoding a putative glycerophosphodiesterphosphodiesterase (GdpD, an enzyme that hydrolyzes PLs) was necessary (but not sufficient) to confer DAP resistance in *E. faecalis* R712 [13]. Therefore, one could hypothesize that the decrease in PG associated with development of DAP resistance in enterococci could be due to the rapid recycling of PG; of note, the glycerol phosphate moiety of PG undergoes rapid turnover in bacteria [26], [29]. It is, thus, tempting to speculate that changes in the “PG pools” are important in the development of DAP resistance by providing the necessary substrates for synthesis of other PLs or glycerol-containing molecules (e.g., lipoteichoic acids) [26], [30] in order to avoid killing by cationic antimicrobial molecules. Of interest, we did not observe any major changes in CL content associated with development of DAP-resistance in *E. faecalis* or *E. faecium* despite the fact that mutations in the CL synthase gene (*cls*) have been previously linked to DAP resistance in *E. faecalis* [16], *E. faecium* [13] and *S. aureus* [14]. Nonetheless, as PG is a substrate for Cls, it is plausible that changes in Cls activity or specificity may also contribute to the shift in PG pools.

*Fourthly*, a significant increase in a negatively charged glycerophosphoglycolipid ultimately identified as GP-DGDAG by LC/ESI-MS/MS was found in both DAP-resistant enterococci studied. It has been previously shown that *E. faecalis* contains several glycolipids including glycerophosphoryl-diglucosyl-diglyceride [31], phosphatidyldiglucosyldiglyceride [32], [33] and glycerophosphoryl-phosphatidylkojibiosyl-diacylglycerol [34]. Interestingly, some of the above glycolipids appear to be the precursors of enterococcal lipoteichoic acids (LTA), which are important constituents of the cell surface of Gram-positive organisms [35]. Indeed, Ganfield & Pieringer showed that PG is the donor of *sn*-glycerol 1-phosphate moieties to phosphatidyl-kojibiosyldiacylglycerol in the *in vitro* synthesis of enterococcal membrane LTA [36]. Furthermore, alanylation of wall teichoic acids (TA) has been shown to play a role in the defense against antimicrobial peptides in *S. aureus* [37] and overexpression of the *dlt* operon, which encodes 4 genes involved in the alanylation of wall TAs in *S. aureus*, has been linked to DAP resistance [38]. Thus, our findings suggest that the emergence of DAP-resistance in enterococci is associated with increase production of membrane glycerophosphoglycolipids which may serve as precursors of LTA using PG as the donor of glycerol-phosphate moiety, which, upon alanylation, might increase relative positive surface charge and contribute to DAP resistance.

*Fifthly*, changes in the fatty acid composition of bacterial CMs are essential in the optimization of CM function for growth and response to modifications in environmental conditions (including the presence of antimicrobial molecules) [26]. Fatty acid composition is also a major determinant of CM order (fluidity-rigidity) that affects the homeostasis of the bacterial cell. In the current study, the development of DAP resistance in both enterococcal isolates was associated with less fluid (more rigid) membranes. Of interest, this is in contrast to previous reports in clinically-derived DAP-resistant *S. aureus* strains, whose CMs were significantly more fluid than their respective DAP-susceptible parental strains [19]. Conversely, a modest decrease in CM fluidity was observed in a DAP-resistant *S. aureus* strain selected by serial *in vitro* passages in DAP [39]. These apparent paradoxes in CM order responses indicate that such CM changes may well be either strain-specific and/or dependent on diverse DAP exposure conditions [40]. Additionally, factors that influence the development of DAP resistance *in vivo* are highly likely to be different from those *in vitro*, including a cadre of host factors such as serum components, host immune cells and innate host defense peptides [39]. Interestingly, the decrease in CM fluidity observed in our DAP-resistant *E. faecalis* strain could not be linked to changes in the proportion of saturated vs unsaturated (or cyclic) fatty acids, and, thus, other factors may be important. In contrast, a significant decrease in unsaturated fatty acids was observed in the DAP-resistant *E. faecium* isolate (mostly due to a decrease in the C18:1ω7c species), representing a potential explanation for the observed reduction in CM fluidity in this strain. It should be noted that there was a significant increase in cyclopropane fatty acids observed in this same DAP-resistant *E. faecium* isolate as compared to its DAP-susceptible parent. Cyclopropane fatty acids have been shown to stabilize CMs to a variety of environmental challenges [41].

In summary, we present evidence that development of DAP resistance in clinical enterococcal strains is associated with a significant shift in PL profiles (i.e., decreases in PG and increases in GP-DGDAG). A common feature of DAP resistance in both *E. faecalis* and *E. faecium* include marked ultrastructural changes in the cell envelope, as well as increases in the net surface positive charge and increased rigidity of the CMs. Our findings suggest that important biochemical and biophysical modifications in CM lipid metabolism occur in response to DAP exposures in enterococci.

## Materials and Methods

### Bacterial isolates and susceptibility testing

The bacterial isolates used in this study are clinical-strain pairs of DAP-susceptible and DAP-resistant *E. faecalis* (S613 and R712, respectively) and *E. faecium* (S447 and R446 respectively); the DAP-resistant derivatives emerged during DAP therapy, and have been described previously [7], [8]. Briefly, the *E. faecalis* pair was recovered from the bloodstream of a patient presenting with recurrent bacteremia in 2005 [7]. The *E. faecium* pair was obtained in 2006 from the urine and blood of a hospitalized patient with acute leukemia [8]. Both clinical strain-pairs exhibited high-level resistance to vancomycin (MIC>128 µg/ml) and the presence of the *vanA* gene was confirmed using a PCR assay as described before [42]. Each strain-pair was utilized for all assays related to phospholipid (PL) analysis, while characterization of the cell envelope was performed only in the *E. faecium* (cell envelope characteristics of the *E. faecalis* pair have been previously reported by us [13]). DAP MICs were performed by Etest on Mueller-Hinton agar following the recommendation of the Clinical Laboratory Standards Institute. In order to confirm the genetic relatedness of each strain pair, pulsed field gel electrophoresis was performed in all isolates following the protocol previously described [3].

### CM PL composition and asymmetry

PLs were extracted from enterococcal isolates using a methodology described before [43], [44]. Briefly, extraction of PLs was performed on enterococcal isolates grown in brain heart infusion (BHI) broth for 18 h (late stationary phase). The major enterococcal PLs were separated using two-dimensional thin-layer chromatography (2D-TLC) using Silica 60 F254 HPTLC plates (Merck). The protocol used first-dimension chloroform-methanol–25% ammonium hydroxide (65∶25∶6, by volume) in the vertical orientation, and a second-dimension chloroform:water:methanol:glacial acetic acid:acetone (45∶4∶8∶9∶16, by volume) in the horizontal orientation for the separation of the PLs and for additional quantitation by phosphate estimation [44]. The identification of individual TLC PL spots was made in comparison to control 2D-TLC plates of known PL standards (**Figure 1**). All PL standards were purchased from Avanti Polar Lipids (Alabaster, AL). Quantitative analysis of individual PLs isolated from TLC plates was performed by digesting with 70% perchloric acid (0.3 mL) at 180°C for 3 h. The PLs were quantified spectrophotometrically at *A*_660_ as described before [44]. The results are reported as the mean (± SD) of at least three independent experiments performed on separate days.

Since the relative outer-to-inner CM asymmetry of PLs can influence the overall surface charge in Gram-positive bacteria [44], the CM distribution of amino-containing (positively-charged) PLs in the outer vs. inner CM bilayers was determined. For this purpose, we used quantitative fluorescamine analysis, since this fluorophore specifically labels only surface-exposed amino-PLs in the outer CM leaflet. Fluorescamine assays for labeling, quantitative estimation of PLs, and chemical analysis of inner vs. outer CM leaflet PLs have been previously described [19], [44]. These latter studies were performed in parallel with the PL analyses above. All PL spots on the TLC plate were identified and confirmed by exposure to iodine vapors and spraying with CuSO4 (100 mg/ml) containing 8% phosphoric acid (v/v) and heated at 180°C [45]. I-LPG (positively charged), and other amino-containing PLs were visualized by ninhydrin staining.

### Liquid chromatography/electrospray ionization-mass spectrometry/mass spectrometry (LC/ESI-MS/MS) analysis

Major lipids separated on the 2D-TLC plate were scrapped off, extracted with chloroform:methanol (1∶1) and analyzed by LC/MS. The major spots observed by 2D-TLC were confirmed by normal phase LC/ESI-MS/MS analysis. Normal phase LC was performed on an Agilent 1200 Quaternary LC system equipped with an Ascentis Silica HPLC column, 5 µm, 25 cm×2.1 mm (Sigma-Aldrich, St. Louis, MO). Mobile phase A consisted of chloroform/methanol/aqueous ammonium hydroxide (800∶195∶5, v/v); mobile phase B consisted of chloroform/methanol/water/aqueous ammonium hydroxide (600∶340∶50∶5, v/v); mobile phase C consisted of chloroform/methanol/water/aqueous ammonium hydroxide (450∶450∶95∶5, v/v). The elution program consisted of the following: 100% mobile phase A was held isocratically for 2 min and then linearly increased to 100% mobile phase B over 14 min and held at 100% B for 11 min. The LC gradient was then changed to 100% mobile phase C over 3 min and held at 100% C for 3 min, and finally returned to 100% A over 0.5 min and held at 100% A for 5 min. The total LC flow rate was 300 µl/min. To achieve optimum ESI efficiency, a post-column splitter was used to divert ∼10% of the LC effluent into the mass spectrometer, a QSTAR XL quadrupole time-of-flight tandem mass spectrometer (Applied Biosystem, Foster City, CA). Instrumental settings for negative ion electrospray (ESI) and MS/MS analysis of lipid species were as follows: IS = −4500 V; CUR = 20 psi; GSI = 20 psi; DP = −55 V; and FP = −150 V. The MS/MS analysis used nitrogen as the collision gas. Data analysis was performed using Analyst QS software (Applied Biosystem, Foster City, CA).

### CM fatty acid composition

Gas-liquid chromatography was used to study the fatty acid composition of total lipids extracted from enterococcal CMs, and analyzed after conversion to their methyl-ester form (using fatty acid standards) as previously described (courtesy of Microbial ID Inc.,Newark, DE) [46].

### CM fluidity

CM fluidity was assessed using the fluorescent probe 1,6-diphenyl-1,3,5-hexatriene (DPH). The protocol followed previously-published techniques for DPH incorporation into target CMs, measurement of fluorescence polarization and calculation of the degree of fluorescence polarization (polarization index) [46]. Excitation and emission wavelengths of DPH are 360 nm and 426 nm, respectively, and were measured using a Biotek Model SFM 25 spectrofluorimeter. The results were interpreted according to the polarization index, since an inverse correlation exists between polarization index values and fluidity (i.e., a lower index equates to a greater extent of CM fluidity [46]). The experiments were conducted at least 3 times for each isolate on separate days.

### Ultrastructural analysis of the cell envelope of the *E. faecium* clinical strain-pair

Comparative visualization of the cell envelope of *E. faecium* S447 and R446 was performed using transmission electron microscopy following standard methodology [13]. Cell wall thickness was evaluated in both isolates by performing 75 separate observations of each isolate (minimum of 50 cells) at 200,000 X magnification in cells from different fields. Thickness of the cell walls of each isolate was measured from the outer border of the CM to the outer edge of the cell wall. The means of cell wall thickness (± SD) were determined for each isolate.

### CM surface charge and DAP-induced CM permeabilization and depolarization

A cytochrome *c* assay [13] was performed to measure the overall relative cell surface positive charge of the clinical *E. faecium* strain-pair following the methodology used before for the *E. faecalis* strain-pair [13]. The amount of cytochrome *c* (a highly positively-charged molecule) remaining in the supernatant after 15 min exposure to each enterococcal strain was determined at *A*_530_. Cytochrome c interacts with the CM in a charge-dependent manner [47]. Thus, the greater the amount of residual supernatant cytochrome *c*, the greater the relative surface positive charge.

DAP-induced CM permeabilization was measured with the LIVE/DEAD BacLight kit which is based on the nucleic acid-specific viability dyes, propidium iodide and SYTO9, as described before [48], [49]. The reaction is based on the observation that viable bacterial cells with an intact plasma membrane are stained by the CM-permeant green fluorescent dye SYTO9. If the membrane is compromised and membrane permeabilization occurs, SYTO9 fluorescence is quenched by entry of propidium iodide into the cytoplasm [13], [49]. SYTO9 fluorescence was measured following excitation at 488 nm and emission at 510 nm.

The CM potential-sensitive 3,3-dipentyoxacarbocyanine [DiSC3(5)] assay [19] was used to measure DAP-induced changes in CM potential in *E. faecium* S447 and R446 as previously described [13]. Fluorescence was measured with an excitation wavelength of 622 nm and an emission wavelength of 670 nm. Loss of red fluorescence indicated CM depolarization.

CM permeabilization and potential were both measured in the presence of increasing concentrations of DAP supplemented with 50 mg/L of calcium chloride in the buffer. A positive control of 100% ethanol and a negative control of buffer alone were included for these two assays. Percent fluorescence change was calculated, setting the ethanol control as 100% fluorescence change and buffer control as 0% fluorescence change. Pilot studies confirmed that there was no spontaneous CM permeabilization or depolarization observed over the study time-periods of these investigations (data not shown).

### Statistical analysis

Differences in cell wall thickness, PL composition and polarization index profiles were compared using a Student's *t*-test. A P value <0.05 was consider significant.

## References

[1] Hidron AI, Edwards JR, Patel J, Horan TC, Sievert DM, et al (2008) NHSN annual update: antimicrobial-resistant pathogens associated with healthcare-associated infections: annual summary of data reported to the National Healthcare Safety Network at the Centers for Disease Control and Prevention, 2006-2007. Infect Control Hosp Epidemiol 29: 996–1011. 18947320 10.1086/591861

[2] Rice LB (2008) Federal funding for the study of antimicrobial resistance in nosocomial pathogens: no ESKAPE. J Infect Dis 197: 1079–1081. 18419525 10.1086/533452

[3] Panesso D, Reyes J, Rincon S, Diaz L, Galloway-Pena J, et al (2010) Molecular epidemiology of vancomycin-resistant *Enterococcus faecium*: a prospective, multicenter study in South American hospitals. J Clin Microbiol 48: 1562–1569. 20220167 10.1128/JCM.02526-09 PMC2863891

[4] Fowler VG Jr, Boucher HW, Corey GR, Abrutyn E, Karchmer AW, et al (2006) Daptomycin versus standard therapy for bacteremia and endocarditis caused by *Staphylococcus aureus* . N Engl J Med 355: 653–665. 16914701 10.1056/NEJMoa053783

[5] Twilla JD, Finch CK, Usery JB, Gelfand MS, Hudson JQ, et al (2011) Vancomycin-resistant Enterococcus bacteremia: An evaluation of treatment with linezolid or daptomycin. J Hosp Med 7: 243–238. 22076962 10.1002/jhm.994

[6] Mave V, Garcia-Diaz J, Islam T, Hasbun R (2009) Vancomycin-resistant enterococcal bacteraemia: is daptomycin as effective as linezolid? J Antimicrob Chemother 64: 175–180. 19423543 10.1093/jac/dkp154

[7] Munoz-Price LS, Lolans K, Quinn JP (2005) Emergence of resistance to daptomycin during treatment of vancomycin-resistant *Enterococcus faecalis* infection. Clin Infect Dis 41: 565–566. 16028170 10.1086/432121

[8] Lewis JS 2nd, Owens A, Cadena J, Sabol K, Patterson JE, et al (2005) Emergence of daptomycin resistance in *Enterococcus faecium* during daptomycin therapy. Antimicrob Agents Chemother 49: 1664–1665. 15793168 10.1128/AAC.49.4.1664-1665.2005 PMC1068653

[9] Green MR, Anasetti C, Sandin RL, Rolfe NE, Greene JN (2006) Development of daptomycin resistance in a bone marrow transplant patient with vancomycin-resistant *Enterococcus durans* . J Oncol Pharm Pract 12: 179–181. 17022872 10.1177/1078155206069165

[10] Silverman JA, Perlmutter NG, Shapiro HM (2003) Correlation of daptomycin bactericidal activity and membrane depolarization in *Staphylococcus aureus* . Antimicrob Agents Chemother 47: 2538–2544. 12878516 10.1128/AAC.47.8.2538-2544.2003 PMC166110

[11] Pogliano J, Pogliano N, Silverman J (2012) Daptomycin mediated reorganization of membrane architecture causes mislocalization of essential cell division proteins. J Bacteriol: In press. 10.1128/JB.00011-12 PMC3415520 22661688

[12] Mehta S, Cuirolo AX, Plata KB, Riosa S, Silverman JA, et al (2012) VraSR two-component regulatory system contributes to mprF-mediated decreased susceptibility to daptomycin in in vivo-selected clinical strains of methicillin-resistant *Staphylococcus aureus* . Antimicrob Agents Chemother 56: 92–102. 21986832 10.1128/AAC.00432-10 PMC3256076

[13] Arias CA, Panesso D, McGrath DM, Qin X, Mojica MF, et al (2011) Genetic basis for in vivo daptomycin resistance in enterococci. N Engl J Med 365: 892–900. 21899450 10.1056/NEJMoa1011138 PMC3205971

[14] Peleg AY, Miyakis S, Ward DV, Earl AM, Rubio A, et al (2012) Whole genome characterization of the mechanisms of daptomycin resistance in clinical and laboratory derived isolates of *Staphylococcus aureus* . PLoS One 7: e28316. 22238576 10.1371/journal.pone.0028316 PMC3253072

[15] Yang SJ, Xiong YQ, Dunman PM, Schrenzel J, Francois P, et al (2009) Regulation of mprF in daptomycin-nonsusceptible *Staphylococcus aureus* strains. Antimicrob Agents Chemother 53: 2636–2637. 19289517 10.1128/AAC.01415-08 PMC2687189

[16] Palmer KL, Daniel A, Hardy C, Silverman J, Gilmore MS (2011) Genetic basis for daptomycin resistance in enterococci. Antimicrobial agents and chemotherapy 55: 3345–3356. 21502617 10.1128/AAC.00207-11 PMC3122436

[17] Hachmann AB, Sevim E, Gaballa A, Popham DL, Antelmann H, et al (2011) Reduction in membrane phosphatidylglycerol content leads to daptomycin resistance in *Bacillus subtilis* . Antimicrob Agents Chemother 55: 4326–4337. 21709092 10.1128/AAC.01819-10 PMC3165287

[18] Cui L, Isii T, Fukuda M, Ochiai T, Neoh HM, et al (2010) An RpoB mutation confers dual heteroresistance to daptomycin and vancomycin in *Staphylococcus aureus* . Antimicrob Agents Chemother 54: 5222–5233. 20837752 10.1128/AAC.00437-10 PMC2981288

[19] Jones T, Yeaman MR, Sakoulas G, Yang SJ, Proctor RA, et al (2008) Failures in clinical treatment of *Staphylococcus aureus* Infection with daptomycin are associated with alterations in surface charge, membrane phospholipid asymmetry, and drug binding. Antimicrob Agents Chemother 52: 269–278. 17954690 10.1128/AAC.00719-07 PMC2223911

[20] Sader HS, Jones RN (2009) Antimicrobial susceptibility of Gram-positive bacteria isolated from US medical centers: results of the Daptomycin Surveillance Program (2007-2008). Diagn Microbiol Infect Dis 65: 158–162. 19748426 10.1016/j.diagmicrobio.2009.06.016

[21] dos Santos Mota JM, den Kamp JA, Verheij HM, van Deenen LL (1970) Phospholipids of *Streptococcus faecalis* . J Bacteriol 104: 611–619. 4321329 10.1128/jb.104.2.611-619.1970 PMC285035

[22] Bao Y, Sakinc T, Laverde D, Wobser D, Benachour A, et al (2012) Role of mprF1 and mprF2 in the pathogenicity of *Enterococcus faecalis* . PLoS ONE 7: e38458. 22723861 10.1371/journal.pone.0038458 PMC3377626

[23] Ernst CM, Staubitz P, Mishra NN, Yang SJ, Hornig G, et al (2009) The bacterial defensin resistance protein MprF consists of separable domains for lipid lysinylation and antimicrobial peptide repulsion. PLoS pathogens 5: e1000660. 19915718 10.1371/journal.ppat.1000660 PMC2774229

[24] Rubio A, Moore J, Varoqlu M, Conrad M, Chu M, et al (2012) LC-MS/MS characterization of phospholipid content in daptomycin-susceptible and -resistant isolates of *Staphylococcus aureus* with mutations in *mprF* . Mol Membr Biol 29: 1–8. 22276671 10.3109/09687688.2011.640948

[25] Sakayori Y, Muramatsu M, Hanada S, Kamagata Y, Kawamoto S, et al (2003) Characterization of *Enterococcus faecium* mutants resistant to mundticin KS, a class IIa bacteriocin. Microbiology 149: 2901–2908. 14523122 10.1099/mic.0.26435-0

[26] Zhang YM, Rock CO (2008) Membrane lipid homeostasis in bacteria. Nat Rev Microbiol 6: 222–233. 18264115 10.1038/nrmicro1839

[27] Zhang W, Campbell HA, King SC, Dowhan W (2005) Phospholipids as determinants of membrane protein topology. Phosphatidylethanolamine is required for the proper topological organization of the gamma-aminobutyric acid permease (GabP) of Escherichia coli. J Biol Chem 280: 26032–26038. 15890647 10.1074/jbc.M504929200

[28] Mileykovskaya E, Dowhan W (2009) Cardiolipin membrane domains in prokaryotes and eukaryotes. Biochim Biophys Acta 1788: 2084–2091. 19371718 10.1016/j.bbamem.2009.04.003 PMC2757463

[29] Goldberg DE, Rumley MK, Kennedy EP (1981) Biosynthesis of membrane-derived oligosaccharides: a periplasmic phosphoglyceroltransferase. Proc Natl Acad Sci U S A 78: 5513–5517. 6272307 10.1073/pnas.78.9.5513 PMC348776

[30] Dowhan W (1997) Molecular basis for membrane phospholipid diversity: why are there so many lipids? Annu Rev Biochem 66: 199–232. 9242906 10.1146/annurev.biochem.66.1.199

[31] Fischer W, Ishizuka I, Landgraf HR, Herrmann J (1973) Glycerophosphoryl diglucosyl diglyceride, a new phosphoglycolipid from Streptococci. Biochim Biophys Acta 296: 527–545. 4347390 10.1016/0005-2760(73)90113-6

[32] Ambron RT, Pieringer RA (1971) The metabolism of glyceride glycolipids. V. Identification of the membrane lipid formed from diglucosyl diglyceride in *Streptococcus faecalis* ATCC 9790 as an acylated derivative of glyceryl phosphoryl diglucosyl glycerol. J Biol Chem 246: 4216–4225. 4326211

[33] Fischer W, Landgraf HR, Herrmann J (1973) Phosphatidyldiglucosyl diglyceride from Streptococci and its relationship to other polar lipids. Biochim Biophys Acta 306: 353–367. 4353702 10.1016/0005-2760(73)90174-4

[34] Fischer W, Landgraf HR (1975) Glycerophosphoryl phosphatidyl kojibiosyl diacylglycerol, a novel phosphoglucolipid from *Streptococcus faecalis* . Biochim Biophys Acta 380: 227–244. 804328 10.1016/0005-2760(75)90009-0

[35] Carson DD, Pieringer RA, Daneo-Moore L (1981) Effect of cerulenin on cellular autolytic activity and lipid metabolism during inhibition of protein synthesis in *Streptococcus faecalis* . J Bacteriol 146: 590–604. 6111555 10.1128/jb.146.2.590-604.1981 PMC217002

[36] Ganfield MC, Pieringer RA (1980) The biosynthesis of nascent membrane lipoteichoic acid of *Streptococcus faecium* (*S. faecalis* ATCC 9790) from phosphatidylkojibiosyl diacylglycerol and phosphatidylglycerol. J Biol Chem 255: 5164–5169. 6768734

[37] Bertsche U, Weidenmaier C, Kuehner D, Yang SJ, Baur S, et al (2011) Correlation of daptomycin resistance in a clinical *Staphylococcus aureus* strain with increased cell wall teichoic acid production and D-alanylation. Antimicrob Agents Chemother 55: 3922–3928. 21606222 10.1128/AAC.01226-10 PMC3147621

[38] Yang SJ, Kreiswirth BN, Sakoulas G, Yeaman MR, Xiong YQ, et al (2009) Enhanced expression of dltABCD is associated with the development of daptomycin nonsusceptibility in a clinical endocarditis isolate of *Staphylococcus aureus* . J Infect Dis 200: 1916–1920. 19919306 10.1086/648473 PMC2779839

[39] Mishra NN, Yang SJ, Sawa A, Rubio A, Nast CC, et al (2009) Analysis of cell membrane characteristics of in vitro-selected daptomycin-resistant strains of methicillin-resistant *Staphylococcus aureus* . Antimicrob Agents Chemother 53: 2312–2318. 19332678 10.1128/AAC.01682-08 PMC2687258

[40] Xiong YQ, Mukhopadhyay K, Yeaman MR, Adler-Moore J, Bayer AS (2005) Functional interrelationships between cell membrane and cell wall in antimicrobial peptide-mediated killing of *Staphylococcus aureus* . Antimicrob Agents Chemother 49: 3114–3121. 16048912 10.1128/AAC.49.8.3114-3121.2005 PMC1196293

[41] Glickman MS, Cox JS, Jacobs WR Jr (2000) A novel mycolic acid cyclopropane synthetase is required for cording, persistence, and virulence of *Mycobacterium tuberculosis* . Mol Cell 5: 717–727. 10882107 10.1016/s1097-2765(00)80250-6

[42] Dutka-Malen S, Evers S, Courvalin P (1995) Detection of glycopeptide resistance genotypes and identification to the species level of clinically relevant enterococci by PCR. J Clin Microbiol 33: 1434. 7615777 10.1128/jcm.33.5.1434-1434.1995 PMC228190

[43] Dixit BL, Gupta CM (1998) Role of the actin cytoskeleton in regulating the outer phosphatidylethanolamine levels in yeast plasma membrane. Eur J Biochem 254: 202–206. 9652415 10.1046/j.1432-1327.1998.2540202.x

[44] Mukhopadhyay K, Whitmire W, Xiong YQ, Molden J, Jones T, et al (2007) In vitro susceptibility of *Staphylococcus aureus* to thrombin-induced platelet microbicidal protein-1 (tPMP-1) is influenced by cell membrane phospholipid composition and asymmetry. Microbiology 153: 1187–1197. 17379728 10.1099/mic.0.2006/003111-0

[45] Tsai M, Ohniwa R, Kato Y, Takeshita S, Ohta T, et al (2011) *Staphylococcus aureus* requires cardiolipin for survival under conditions of high salinity. BMC microbiology 11: 13. 21241511 10.1186/1471-2180-11-13 PMC3030509

[46] Bayer AS, Prasad R, Chandra J, Koul A, Smriti M, et al (2000) In vitro resistance of *Staphylococcus aureus* to thrombin-induced platelet microbicidal protein is associated with alterations in cytoplasmic membrane fluidity. Infect Immun 68: 3548–3553. 10816510 10.1128/iai.68.6.3548-3553.2000 PMC97641

[47] Peschel A, Otto M, Jack RW, Kalbacher H, Jung G, et al (1999) Inactivation of the dlt operon in *Staphylococcus aureus* confers sensitivity to defensins, protegrins, and other antimicrobial peptides. J Biol Chem 274: 8405–8410. 10085071 10.1074/jbc.274.13.8405

[48] Leuko S, Legat A, Fendrihan S, Stan-Lotter H (2004) Evaluation of the LIVE/DEAD BacLight kit for detection of extremophilic archaea and visualization of microorganisms in environmental hypersaline samples. Appl Environ Microbiol 70: 6884–6886. 15528557 10.1128/AEM.70.11.6884-6886.2004 PMC525124

[49] Higgins DL, Chang R, Debabov DV, Leung J, Wu T, et al (2005) Telavancin, a multifunctional lipoglycopeptide, disrupts both cell wall synthesis and cell membrane integrity in methicillin-resistant *Staphylococcus aureus* . Antimicrobial agents and chemotherapy 49: 1127–1134. 15728913 10.1128/AAC.49.3.1127-1134.2005 PMC549257

